# Applications and Achievements of Single-Cell Sequencing in Gastrointestinal Cancer

**DOI:** 10.3389/fonc.2022.905571

**Published:** 2022-06-16

**Authors:** Zhenliang Xie, Jincheng Li, Pu Huang, Ye Zhang, Jingkuan Yang, Kangdong Liu, Yanan Jiang

**Affiliations:** ^1^The Pathophysiology Department, School of Basic Medical Sciences, College of Medicine, Zhengzhou University, Zhengzhou, China; ^2^China-US (Henan) Hormel Cancer Institute, Zhengzhou, China; ^3^State Key Laboratory of Esophageal Cancer Prevention and Treatment, Zhengzhou, China; ^4^Basic Medicine Sciences Research Center, Zhengzhou University, Zhengzhou, China; ^5^Provincial Cooperative Innovation Center for Cancer Chemoprevention, Zhengzhou University, Zhengzhou, China; ^6^Cancer Chemoprevention International Collaboration Laboratory, Zhengzhou, China

**Keywords:** single-cell sequencing, gastrointestinal cancer, gastric cancer, esophageal cancer, colon cancer

## Abstract

Gastrointestinal cancer represents a public health concern that seriously endangers human health. The emerging single-cell sequencing (SCS) technologies are different from the large-scale sequencing technologies which provide inaccurate data. SCS is a powerful tool for deciphering the single-cell resolutions of cellular and molecular landscapes, revealing the features of single-cell genomes, transcriptomes, and epigenomes. Recently, SCS has been applied in the field of gastrointestinal cancer research for clarifying the origin and heterogeneity of gastrointestinal cancer, acquiring micro-environmental information, and improving diagnostic and treatment methods. This review outlines the applications of SCS in gastrointestinal cancer research and summarizes the most recent advances in the field.

## Introduction

**Figure 1:**
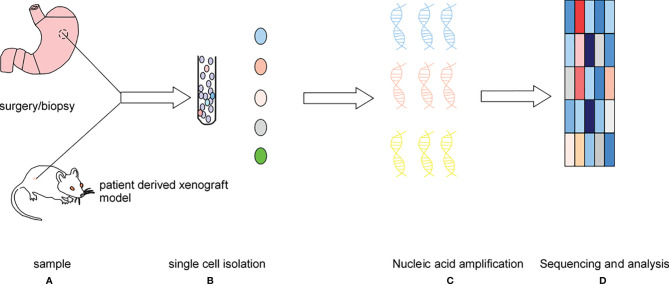
The main processes: **(A)** sample acquisition, **(B)** cell isolation, **(C)** single-cell DNA or RNA amplification, **(D)** high-throughput sequencing and single-cell sequencing data correction and analysis.

Single-cell sequencing (SCS) has rapidly developed in recent years. The first single-cell mRNA sequencing experiment was performed in 2009, then the first single-cell DNA sequencing experiment in human cancer cells was performed two years later, and the first single-cell exon sequencing experiment was performed in 2012. In 2013, Picelli et al. made some improvements on the smart-seq technology, an SCS protocol (1). This new technology is called smart-seq2. In 2017, 10xGenomics and the Fred Hutchinson Cancer Research Center developed a new single-cell RNA sequencing (scRNA-seq) method, and since then, thousands of immune cells had been analyzed (2, 3). Single-cell sequencing involves the isolation of the cell group in the tissue or body fluid to a single cell level, then the expansion of the extracted nucleic acid (DNA or RNA) to the lowest detection level, followed by sequencing of the genome and transcriptome, and finally correction and analysis of the data. The SCS procedure is shown in **Figure 1**.

Gastrointestinal (GI) cancers are one of the most common malignant tumors and comprise gastric cancer (GC), esophageal cancer (EC), colorectal cancer (CRC), pancreatic cancer, etc. They are characterized by high morbidity, mortality, malignancy rates, and rapid development (4). GC has a high global prevalence. It accounted for more than 1 million new cases and an estimated 769,000 deaths in 2020, ranking fifth among the causes of global cancer morbidity and fourth among those of cancer mortality (5). Due to the lack of obvious early symptoms, the mortality rate of GC was still one of the highest among malignant tumors. Intriguingly, the incidence of EC was ranked seventh (604,000 cases) for new cases and sixth in the overall mortality rate (544,000 deaths) in 2020. Moreover, CRC (including anal cancer), which accounted for over 1.9 million new cases and 935,000 deaths, ranked third in terms of incidence, but second in terms of mortality among all cancers in 2020. GI cancer is characterized by complex heterogeneity (6) and a specific tumor microenvironment and is extremely suitable for promoting tumor progression and metastasis (7).

**Figure 2:**
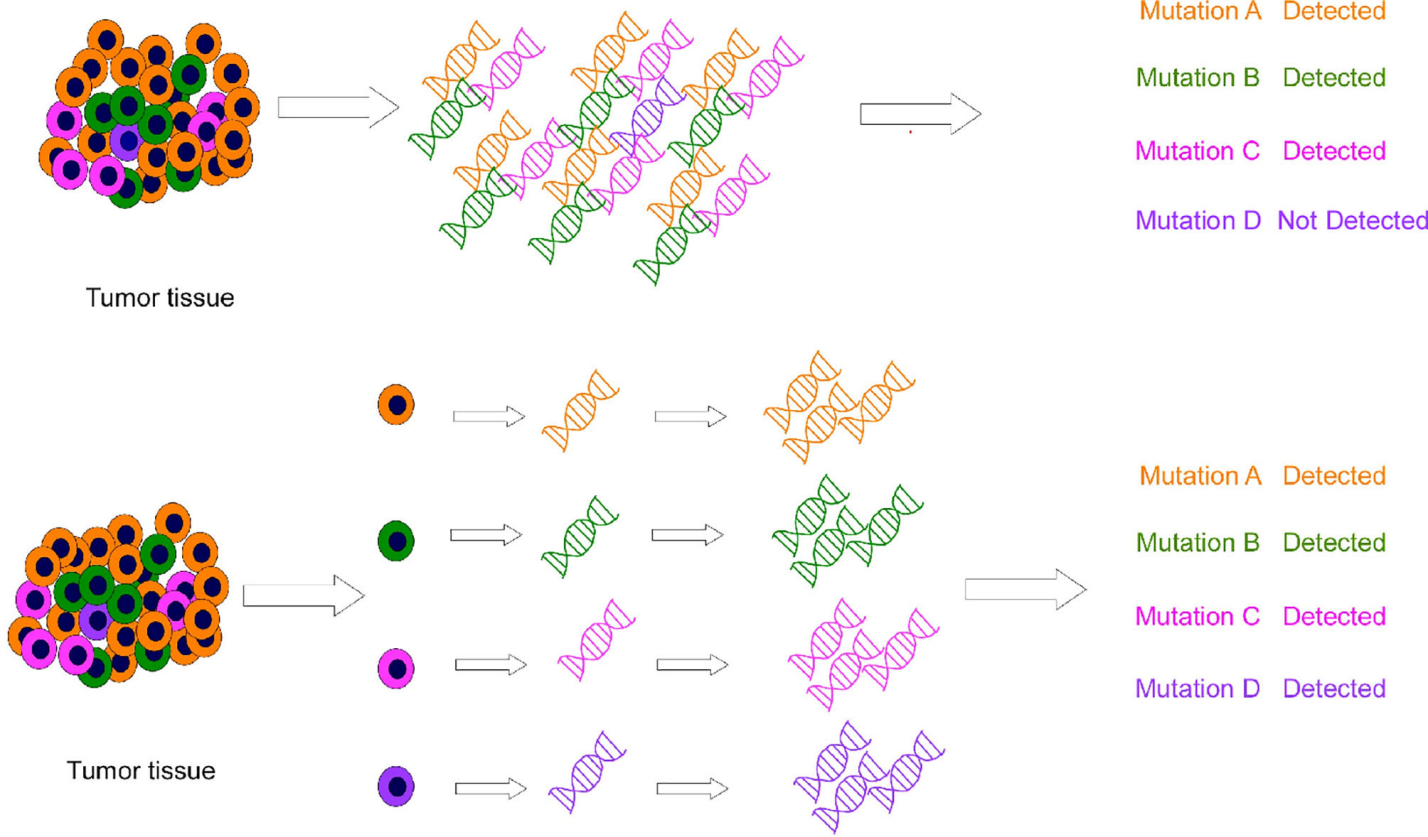
Conventional sequencing (methods above) results in the neglect of some low-abundance information, and single-cell sequencing (methods below) combines cell heterogeneity.

SCS plays a significant role in cancer research. Bulk sequencing does not perform well with intratumoral heterogeneity, as it misses rare mutations. For example, in cancer cells, mutations are diluted or lost during averaging of the bulk sequencing (8). In contrast, SCS can be used for the molecular profiling of individual cells and helps in obtaining more precise information about the tumor (**Figure 2**). Therefore, SCS is a potential superior alternative to traditional sequencing methods. Moreover, although the combination of new resistant chemotherapy, molecular targeted therapy, and immunotherapy techniques has shown promising anti-tumor effects against advanced GI tumors, these techniques have several limitations. SCS can better help researchers investigate problems in tumor heterogeneity, microenvironment, diagnosis and treatment. Based on these advantages, many researchers have made important achievements in cancer research by using this technique. This review focuses on SCS and its applications and achievements in GI cancer studies.

## SCS Applications and Achievements in GC

### SCS Reveals the Origin and Heterogeneity of GC

Tumor heterogeneity exists due to cell groups with different genotypes in tumor cells during the process of growth, this different cell groups lead to phenotypic inconsistencies. Intratumoral heterogeneity which is observed in various cancers is also one of the main clinical and pathological characteristics of GC. Cellular heterogeneity is significant genotypic differences within the same phenotype and leads to the differences in the growth, invasion, metastasis and drug sensitivity of GC cells (9, 10). Many researchers have used SCS to investigate the origin and heterogeneity of GC (**Table 1**). Andor et al. characterized cell diversity in nine GC cell lines and assessed gastric cancer heterogeneity and detected scarce clones more efficiently by the high resolution of SCS (11). Peng et al. identified 201 individual cell single nucleotide variations, including 117 non-synonymous mutations, in GC cells by using SCS. They also revealed that there were 24 significant mutant genes, such as *CTAGE5, REC8, SORD, and PTCH2* genes, in single cells, wherein the change in single amino acids affected protein conformation. This study firstly showed the mutation pattern of GC cells at the intratumoral level and provided more important information for understanding individualized targeted therapy and the heterogeneity of GC cancer (12). Based on the SCS results of three primary and paired metastatic lymph node cancers in GC patients, Wang et al. identified different tumor characteristics and different patients with different microenvironment subsets; moreover, their clustering data revealed that *KIF5B, NOTCH2, NOTCH2NL* and *ERBB4* were highly expressed in primary carcinomas, whereas *ERBB2, CDK12*, and *CLDN11* were highly expressed in metastatic carcinomas (13). Similarly, in Zhang’s experiment, they classified a subclass of tumor-specific epithelial cells as “GC type 1”, and a subclass consisting of epithelial cells and normal tissues of GC as “GC type 2”. The expression of the intestinal mucosal markers *MUC13, TFF3, SPINK4*, *FABP1*, and *REG4* were increased in the GC type 1 subclass, while, the expression of the previously identified gastric cancer marker gene *KRT7* was increased significantly in the GC type 2 subclass (14). A previous study used SCS and GC tumor cell clusters (C1-C5) to investigate that *REG4, CLDN4, TFF3*, and *CLDN7* were upregulated in the malignant epithelium as compared with that in the non-malignant epithelium. In addition, *PGC, MUC5AC, LIPF*, and *GKN1* were highly expressed in the non-malignant epithelium (15). In a recent study, Wang et al. clarified the relationship between tumor cell lineage/state composition and intratumoral heterogeneity at the transcriptional, genotypic, molecular, and phenotypic levels by using SCS. The study demonstrated the diversity of tumor cell lineage/state components in peritoneal carcinomatosis (PC) samples. The relationship was defined as the key factor responsible for intratumoral heterogeneity (16).

**Table 1:** SCS in gastric cancer heterogeneity.

Sample	Single Cell isolation	Molecular level	Amplification	Achievement	Reference
Gastric cancer cell lines	Flowmi cell strainer	DNA and RNA	Droplet-based reagent delivery system	Demonstrated a new scDNA-seq technology combined with scRNA-seq and enabled more accurate interrogation of intratumoral heterogeneity.	(11)
Tumor tissue of a patients	Agilent SureSelect Platform	DNA	Agilent SureSelect Platform	Revealed 24 significant mutant genes (SMGs) identified in single cell(*SORD,REXO2,REC8,PTCH2,CTAGE5,RNF20,RBBP4,MGAT4A,KIF15,XYLT1,IGF2BP3,DLC1,TG,CDC27,BAZ1A,ETV6,FLG,NEK6,NSD1,PDE4DIP,RAF1,RNF2,SMO,ZNF483*). The mutant genes CDC27 and FLG might alter the protein conformation only in single cell but not in the corresponding tumor tissue.	(12)
Tumor tissue from 3 patients	Fluorescence microscopy	RNA	Smart-seq2	Discovered some GC lymph node metastasis marker genes (*ERBB2, CLDN11* and *CDK12*), as well as potential gastric cancer evolution−driving genes (*FOS* and *JUN*). *ERBB4, NOTCH2, KIF5B* and *NOTCH2NL* were highly expressed in primary cancer, while *CDK12, CLDN11* and *ERBB2* were over expressed in metastatic cancer.	(13)
7 patients with GC and one patient with gastrointestinal metaplasia	10x Genomics	RNA DNA	10x Genomics	The transcriptional activation levels of GC1 and GC2 cells with different carcinogenic pathways were significantly different.	(14)
9 tumor and 3 non- tumor tissue.	FACS 10x Genomics	RNA	10x Genomics	Uncovered high intratumour differentiation heterogeneity in patients such as IGC1 and IGC4. Several genes specifically expressed in C4 cluster, *RNF43 , GGH, BMP4, DPEP1*.	(15)
15 patients with gastric adenocarcinoma	10x Genomics	RNA	10x Genomics	Tumor cells were divided into four clusters. Cells within C4 expressed the highest levels of entero-derived marker genes, such as *DMBT1, FCGBP, PIGR*, and *WFDC2*, whereas cells within C1 had the highest levels of marker genes expression, such as PSCA and TFF1.	(16)

### SCS Enables the Discovery of the Features of GC Microenvironment

Tumor cells and their microenvironments are interactive and coevolutionary. The tumor immune microenvironment comprises various tumor-infiltrating immune cells (such as B lymphocytes, T lymphocytes, mast cells, natural killer cells, and myeloid suppressor cells) (17, 18). Tumor cells are also surrounded by the stroma, which is divided into cellular and acellular parts. These compartments are composed of complex tumor microenvironments that interact with cancer cells. SCS helps to clarify the molecular level mechanism of the immune cells in the tumor microenvironment during tumor cell generation, development, metastasis, drug resistance and immune escape. It contributes to a more accurate clinical diagnosis, treatment, and prognosis of solid tumors (19, 20). In the past few years, several researchers have made significant advances in microenvironmental research by using SCS to analyze GC cells (**Table 2**). For example, Eum et al. used SCS and found that macrophages which were recovered from malignant ascites of GC patients have non-inflammatory characteristics and the anti-inflammatory properties of tumor-associated macrophages (TAMs) were controlled by tumor cells. These findings helped the researchers to continuously improve the treatment strategies for patients with GC (21). Another study by Meyer et al. demonstrated that the expression of several secretory factors, including IL4, IL5, IL9, IL13, and ARG, were involved in the function of L635-treated ILC2 (type 2 innate lymphoid) cells (22). Fu et al. found that the expression of the transcription factor IRF8 in CD8+ tumor-infiltrating lymphocytes in GC tissue was downregulated in the late stage of GC the disease by using SCS. These findings provided a further rationale for targeted immunotherapy in GC (23). Through SCS analysis, researchers confirmed that tumor-specific macrophages existed in a continuum of stimulus-dependent functional states and were regulated by a specific set of genes. They even found that the increase of the abundance of regulatory cells (Tregs) in the gastric tumor microenvironment was related to immunosuppression (14). Kwon et al. found that the dynamic tumor evolution was more related to the collapse of mutant structures in treatment response. Different T-cell receptor lineages were found to be related with a longer progression-free survival with pembrolizumab treatment by combining whole-exome sequencing (WES) with scRNA-seq. In addition, the increase in the number of PD-1^+^CD8^+^ T cells was associated with lasting clinical benefits (24).

**Table 2:** SCS in microenvironment of gastric cancer.

Sample	Single Cell isolation	Molecular level	Amplification	Achievement	Reference
7 patients with GC and one patient with gastrointestinal metaplasia	10x Genomics	RNA DNA	10x Genomics	Cyclin B1 was upregulated by the loss of *CDC27*. *CDC27* was a tumor suppressor inactivated after the mutation.	(14)
15 patients with gastric adenocarcinoma	10x Genomics	RNA	10x Genomics	In tumors with mixed gastrointestinal characteristics, the abundance fraction of B cells increased significantly, with a higher proportion of M1-like macrophages (pro-inflammatory) and a lower proportion of M2-like macrophages (anti-inflammatory).	(16)
Tumor tissue of 4 patients	C1 microfluidic system	RNA	SMART-Seq2	TAMs from GC abundantly expressed proinflammatory cytokines and the macrophages were M2 macrophages.	(21)
Carcinogen- induced mouse model	FACS	RNA	SMART-seq2	ILC2-derived factors were required for the reprogramming of the gastric mucosa after injury and ILC2s performed a central role in the coordination of gastric epithelial repair after severe damage.	(22)
Immune cells in 9 patients with gastric cancer	FACS 10×Genomics	RNA	10×Genomics	*IRF8* was associated with depleted CD8^+^ T cells in the GC. The transcription factor RBPJ was overexpressed in the tumor-infiltrating Tregs. DC cells expressed more inhibitory receptors in GC tissues, for example, FTL and IL8.	(23)
19 patients with metastatic gastric cancer	10× Genomics	RNA	10× Genomics	Patients who showed a good response to pembrolizumab demonstrated abundant preexisting tumor-infiltrating lymphocytes , a diverse pretreatment TCR repertoire, and a high proportion of stem-like exhausted cells in dysfunctional CD8+ TILs.	(24)

### SCS Facilitates the Diagnosis and Treatment of GC

ScRNA-seq approaches can identify optimal combination therapies that efficiently target heterogeneous cell populations. Furthermore, scRNA-seq can identify the alterations associated with treatment resistance in different cell clusters to support individualized cancer therapy (25). The optimization of existing chemotherapeutic agents and the development of targeted therapies have provided more options for the treatment of advanced gastric cancer and further prolonged the survival expectations of the patients. In addition, global efforts, including the employment of SCS, have been made to identify new specific, predictive, sensitive, and prognostic biomarkers and to establish innovative molecular classifications based on gene expression profiles (26). With the use of SCS, the researchers have made great progress in the diagnosis and treatment of gastric cancer (**Table 3**). For example, Zhang et al. used SCS to construct a single-cell network based on the cellular and molecular characteristics of gastric epithelial cells with different lesions and establish OR51E1 as a unique endocrine cell marker in early malignant lesions. They also suggested that HES6 may mark goblet precell clusters and these findings helped to identify metaplasia in the early stage. Zhang et al. also determined the specific characteristics which is clinically significant for its accurate diagnosis in early GC (27). SCS can also help to identify markers related to tumor diagnosis and personalized therapy (33, 34). Wang et al. performed SCS to classify PC samples into two subtypes that were predicted independent of clinical variables, obtained and verified the prognostic markers of 12 genes (*TPM2, FCGBP, CDK6, NCBP2, CLCX3, PIGR, BTF3, CKB, VPS28, TM4SF1, EIF3E, GPX4*) in multiple large-scale gastric adenocarcinoma (GAC) cohorts (16). Another study by Bockerstett et al. established that in Spasmolytic polypeptide-expressing metaplasia (SPEM) and cervical cell proliferation hypertrophy, the expression of SPEM-related transcripts were similar, and the mechanism of drug-mediated parietal cell ablation was similar to that of SPEM induced by chronic inflammation (28). Chen performed single-cell DNA sequencing of 50 target circulating tumor cells (CTCs) and discovered that large multiploid CTCs (LCTCsmulti) and small CTCs with trisomy 8 (SCTCstri) had different gene variations. Moreover, mutations in the KRAS and Rap1 pathways were abundant in SCTCstri, while several unique mutations in the MET/PI3K/AKT pathway and *SMARCB1* genes were found in LCTCsmulti. These findings highlighted the different mechanisms of drug resistance for modulating target therapy and could help in preventing the poor prognosis of patients (29). Based on the data from scRNA-seq, some researchers analyzed GC samples to classify them into three GC differentiation-related genes molecular subtypes. They found that molecular typing based on cell differentiation could successfully predict the overall survival of the patient, immune checkpoint gene expression, clinicopathological features. This study emphasized the significance of GC cell differentiation in predicting the clinical outcomes and potential immunotherapy responses of patients (30). Furthermore, by transplanting two GC cell lines into mice and performing single-cell transcriptome sequencing of the transplanted tumors, Nagaoka confirmed that interleukin-17 (IL-17) could be a potential target for enhanced programmed cell death 1, anti-PD-1 (programmed cell death protein 1) mAb treatment for GC (31). Bockerstett et al. sequenced the transcriptome of gastric mucosal epithelial cells and found that gastrin 3 mRNA was a tumor-specific marker of the gastric epithelium of intestinal metaplasia (32). Analysis of the SCS data from mice with hereditary diffuse gastric cancer (HDGC) revealed that inactivation of Cdh1 led to metastasis along the squamous cell differentiation trajectory associated with aberrant expression of GI epithelial differentiation center genes. Cytokeratin 7 encoded by the differentiation-dependent gene Krt7, was a specific marker of early neoplastic lesions in CDH1 carriers (20). In conclusion, SCS is valuable in identifying prognostic tumor markers for predicting potential clinical outcomes and immune responses, as well as, for individualized therapy.

**Table 3:** SCS in diagnosis and treatment of gastric cancer.

Sample	Single Cell isolation	Molecular level	Amplification	Achievement	Reference
15 patients with gastric adenocar- cinoma	10x Genomics	RNA	10x Genomics	*TPM2, FCGBP, CDK6, NCBP2, CLCX3, PIGR, BTF3, CKB, VPS28, TM4SF1, EIF3E, GPX4* were the prognostic markers in multiple large-scale gastric adenocarcinoma.	(16)
Tumor tissue of 4 patients	C1 microfluidic system	RNA	SMART-Seq2	Combination therapies targeting cancer cells and macrophages might have mutually synergistic effects. *CITED2* alone had prognostic value in predicting overall GC survival by representing macrophage M2 properties.	(21)
Tumor tissue of 13 patients	10x Chro- mium platform	RNA	10x Chro- mium platform	Among the genes upregulated in the endocrine cells of the EGC lesions, *OR51E1* was ranked at the top. *HES6* could be used to label cells with some goblet cell features but had not been morphologically identified as goblet cells.	(27)
Carcinogen-induced mouse model	10x Genomics	RNA	10x Genomics	Muc6 + Gif + epithelial cells were present in healthy stomachs, but did not express SPEM transcripts such as *Tff2, Cd44*, and *Cftr*.	(28)
Tumor tissue of 111 advanced GC patients	NMSCM, Cytelligen	DNA	Single Cell WGA Kit	*V7A* and *G15S* non-synonymous mutations were more frequently identified in SCTCstri, and high frequency of *MET E1214A, PIK3CA K440N FGFR1 M2761,E1214D, K1215E, L687I* and *K1215 N* mutations were detected in LCTCsmulti.	(29)
402 cells from 6 patients	From the GSE112302 dataset in the Gene Expression Omnibus	From the GSE112302 dataset in the Gene Expression Omnibus	From the GSE112302 dataset in the Gene Expression Omnibus	A prognostic risk scoring signature consisting of 8 GC differentiation-related genes was generated(*VCAN, TNFAIP2, STMN2, RNASE1, DUSP1, AQP2, ADAM8, TFF1*).	(30)
Two gastric cancer cell lines, YTN16 and YTN2 were inoculated in C57BL/6 mice	FACS 10X Genomics	RNA	10X Genomics	The combination of anti-IL-17 and anti-PD-1 mAb caused strong tumor regression and was confirmed in a murine gastric cancer model.	(31)
Carcinogen-induced mouse model	10X Genomics	RNA	10X Genomics	Expanded the definition of gastric metaplasia to include Gkn3 mRNA and GKN3-positive cells in the corpus, allowing a more accurate assessment of SPEM.	(32)
Carcinogen-induced mouse model	10X Genomics	RNA	10X Genomics	Cytokeratin 7, encoded by the differentiation-dependent gene *Krt7*, was a specific marker for early neoplastic lesions.	(20)

## SCS Applications and Achievements in EC

### SCS Reveals the Origin and Heterogeneity of EC

SCS plays an important role in studying the origin and heterogeneity of esophageal cancer. Many researchers have used this technique to achieve important results in revealing the esophageal cancer heterogeneity (**Table 4**). For example, Zhang et al. screened the ESCC cells into 38 subsets, and found that there were 24 subsets with more than 75% of the cells coming from an individual patient. These 24 subsets were defined as the cluster 1, and the remained 14 subsets were defined as the cluster 2. Cluster 1 showed increased pathway activity in cell proliferation and EMT, while cluster 2 showed activation of immune-related pathways. This study showed high heterogeneity in ESCC (35). Wu et al. found that NOTCH signaling was not activated in esophageal adenocarcinoma (EAC) and only activated in ESCC. ESCC tumors with higher NOTCH activity were associated with significantly worse survival. Furthermore, levels of *DLL1, JAG1* and *NOTCH2* were higher in ESCC and EAC tumors than in normal tissues, while products of active signature genes (*SNAI2* and *TNFSF10*) were only detected in ESCC tissues. This study revealed differences in cellular transcriptomic profiles of ESCC and EAC, and a wide range of intratumoral cellular heterogeneity. This founding had important implications for future therapeutic strategies and drug development (36). Chen et al. reclustered malignant cells from five ESCC tumor samples and identified five subsets. Each subset was corresponded to a patient. They revealed high-risk genes in different patients, and different patients showed different expression patterns for different high-level genes. Furthermore, the SCS and copy number variations (CNVs) analysis data showed relative changes in the CNV profiles of all tumor cells compared to non-malignant epithelial cells. The tumor cells in each subpopulation exhibited different CNV status. These findings demonstrated the high heterogeneity of ESCC tumor cells in terms of gene expression and CNV status (37). Another study explained the high degree of heterogeneity in the ESCC microenvironment. Macrophages were clustered into five subsets. Among the five macrophage subsets, Mac_1, Mac_2 and Mac_3 expressed higher anti-inflammatory “M2” -related genes in Mac_C, with Mac_5 expressing M2-like genes (38).

**Table 4:** SCS in esophageal cancer heterogeneity.

Sample	Single cell isolation	Molecular level	Amplification	Achievement	Reference
60 ESCC tumor and 4 adjacent normal tissue 60 patients	10x Genomics	RNA	10x Genomics	The quantitative data showed that in patients with the same level of intratumoral heterogeneity, patients with group 1 cluster had relatively higher levels of intertumor heterogeneity than patients without group cluster 1.	(35)
368 single cells from three ESCC and two EAC	10X Genomics	RNA	SMART-seq2	The three ESCC tumors contained an overwhelming majority of cancer cells with notable *TP63/SOX2* over-amplification, which was not apparent in EAC cancer cells.	(36)
5 ESCC patients and 5 corresponding non-malignant patients	10X Genomics	RNA	10X Genomics	*EGR1* was highly expressed in patient 2, whereas S100A8 / 9 was found to be a high-risk ESCC gene in patient 4. Compared to other sub-clus-ters, cluster 1 demonstrated an apparent CNV loss in chromosome 4 and chromosome 5.	(37)
11 patients with ESCC	10X Genomics	RNA	10X Genomics	Mac_1 expressed multiple chemokines,Mac_2 expressed Cathepsin genes,Mac_3 intriguingly expressed a number of nonclassical monocytic genes,Mac_5 was characterized by its specific expression of interferonstimulated genes.	(38)

### SCS Enables the Discovery of the Features of EC Microenvironment

SCS plays an important role in determining the cellular characteristics of the EC microenvironment. To date, various studies have utilized SCS techniques to explore the problems in the microenvironment of EC (**Table 5**). For example, some researchers discovered that TAMs expressed not only genes related to immunosuppression (*TGFB1* and *COX2*) but also genes related to angiogenesis (*VEGFA, CXCL8, MMP9*, and *MMP12*), and found that *VEGFA* was upregulated in monocytes, while MMPs were mainly expressed in TAMs. These results revealed the immunosuppressive status of the esophageal squamous cell carcinoma (ESCC) tumor microenvironment and improved our understanding of ESCC (35). Similarly, several characteristics of CD4^+^ T cells had been identified by using SCS. Three classes of CD4+ T cells were identified *via* SCS, and it was found that CD4_1 upregulates TIGIT expression in tumors, CD4_2 expresses PD-1 (encoded by PDCD1) exclusively in tumors, and CD4_3 showed tumor-specific TIGIT and CD96 expression. In addition, both CD4_1 and CD4_2 were found to express high levels of CTLA-4 in tumors (38). Wu et al. recently showed that cell cycle signaling was associated with high cancer stemness of EAC, such as *E2F3, CHEK1, CDC20, SMC3, and TFDP1*. In addition, they identified a novel cancer stem cell-associated gene, poly (ADP-ribose) polymerase 4, and they validated its association with survival in a cohort study of 121 ESCC patients (39). Another study discovered a strong correlation between FGF2 and SPRY1 expression in EC using SCS. In the fibroblasts in EC tissues, a high FGF2 expression was found to be associated with low overall survival, and the mouse tumor model confirmed that FGF2 overexpression in fibroblasts significantly upregulated SPRY1 expression in the depleted T cells, weakened the cytotoxic activity of T cells, and promoted tumor growth (40). Together, several studies have identified the features of different cells in the EC microenvironment and found some exclusively expressed genes using SCS. These researches has considerably improved our understanding of esophageal carcinogenesis.

**Table 5:** SCS in microenvironment of esophageal cancer.

Sample	Single cell isolation	Molecular level	Amplification	Achievement	Reference
60 ESCC tumor and 4 adjacent normal tissue	10x Genomics	RNA	10x Genomics	Most of TEX cells were likely tumor-reactive T cells. Esophageal squamous carcinoma tissue was enriched in Treg and Tex cells, whereas TN, TMEM, and Teff cells were fewer, suggesting that TME was in an immunosuppressive state	(35)
Primary tumors and matched adjacent nonmalignant esophageal tissues from 11 treatment-naive ESCC patients	10x Genomics	RNA	10x Genomics	CD4_1 expressed abundant follicular-assisted T (TFH) effector genes (*CXCL13, IL2RA, TNFRSF18, TNFRSF)* CD4_2 subset was characterized by effector memory genes, including *IL7R*, and *CXCR6*.	(38)
From the SRA (https://www.ncbi.nlm.nih.gov/sra) under the accession no. SRP119465	From the SRA (https://www.ncbi.nlm.nih.gov/sra) under the accession no.SRP119465	RNA	From the SRA (https://www.ncbi.nlm.nih.gov/sra) under the accession no. SRP119465	Cell cycle signaling was associated with high cancer stemness of EAC,such as *E2F3,CHEK1, CDC20,SMC3 ,TFDP1*.	(39)
8 treatment-naïve ESCC patients	FACS	RNA	10x Genomics	*FGF2* as an important regulator of SPRY1 expression was involved in establishing the dysfunctional state of CD8+ T cells in esophageal cancer.	(40)

### SCS Facilitates the Diagnosis and Treatment of EC

SCS also plays an important role in the diagnosis and treatment of EC by facilitating the identification of diagnostic markers and development of new treatment. Many investigators have applied SCS to make great progress in the diagnosis and treatment of EC (**Table 6**). For example, using scRNA-seq and real-time quantitative PCR, Zhang et al. verified that *RAD51AP1, KIF2C, KIF20A,NUF2,PBK*, and *DEPDC1* are all potential biomarkers for the diagnosis and prognosis of ESCC and may be potential therapeutic targets for ESCC (41). Furthermore, researchers found that CD4^+^ Tregs expressed the highest levels of IL-32 and had relatively low expression in proliferating natural killer cells and suggested that IL-32 may be a target for immunosuppressive therapy in EC (42). Using scRNA-seq analysis of KYSE-30 cells and paclitaxel-resistant KYSE-30 cells (paclitaxel-R), Wu et al. showed that the two subsets based on *KRT19* expression levels had different paclitaxel sensitivity, suggesting there were intrinsic paclitaxel resistance in KYSE-30 cells. They also found that the proteasome inhibitor carfilzomib could attenuate resistance to paclitaxel-R cancer cells by activating HIF-1 signaling, suggesting that the combination of carfilzomib and paclitaxel could be used as a novel cancer treatment (43). Yang et al. carried out scRNA-seq of KYSE-180 cells and found radioresistance in KYSE-180 cells after fractionated irradiation (FIR) treatment. These foundings provided an important reference for developing radiotherapy strategies (44). Another study involved the identification of 42 recurrent radioresponsive genes (sensitive and resistant), including *GAS2L2*, *NOTCH1*, *OBSCN, MAML3*, *NFE2L2,TP53* and *CDKN2A.* This finding provided a reference for the diagnosis and treatment of EC (45).

**Table 6:** SCS in diagnosis and treatment of esophageal cancer.

Sample	Single cell isolation	Molecular level	Amplification	Achievement	Reference
3 ESCC patients and 208 single cells	From the Sequence Read Archive (https://www.ncbi.nlm.nih.gov/sra)	RNA	From the Sequence Read Archive (https://www.ncbi.nlm.nih.gov/sra)	*RAD51AP1, KIF2C, KIF20A, NUF2 , PBK*, and *DEPDC1* were associated with the diagnosis and prognosis of ESCC.	(41)
7 ESCC tumor and paired adjacent tissues of the patient	10 × Genomics	RNA	10 × Genomics	IL-32 was overexpressed in T and NK Cells in the TME. IL-32 was dominant in CD4+ Treg Cells.	(42)
Carcinogen- induced mouse model	FACS	RNA	SMART-seq2	Low HIF-1 and high proteasome expression were critical for acquired paclitaxel resistance in ESCC.	(43)
KYSE- 180 cells	FACS	RNA	SMART-seq2	The *CFLAR, LAMA5, ITGA6, ITGB4*, and *SDC4* genes were verified as radioresistance genes.	(44)
Tumor tissue of 2 patients	Qiagen	DNA	REPLI-g UltraFast Mini Kit	A subset of sensitive mutations in 10 genes and resistant mutations in 18 genes defined a significantly improved prognosis and the shortest time for locoregional recurrence, respectively, indicating possible clinical utility.	(45)

## SCS Applications and Achievements in CRC

### SCS Reveals the Origin and Heterogeneity of CRC

SCS is important in revealing the origin and heterogeneity of CRC. With the help of SCS, many investigators have further revealed the origin and heterogeneity of CRC (**Table 7**). Some researchers performed single-cell analysis of colon cancer samples using high-throughput SCS based on multiple displacement amplification. They focused on a mutant gene, *SLC12A5*, and found that *SLC12A5* activation could promote cell proliferation and inhibit apoptosis, thus potentially promoting oncogenesis and demonstrating the biclonal origin of CRC cases (46). In addition, two normal or adenomatous polyps in CRC patients were studied *via* single-cell whole-exome sequencing and matched bulk WES. The results indicated that accumulation of non-random somatic gene mutations were involved in the GPCR, PI3K-Akt, and *FGFR* signaling pathways were also observed. These new driver mutations in *OR1B1* (GPCR signaling), *LAMA1* (PI3K-Akt signal in CRC evolution), and *ADCY3* (FGFR signaling) of adenoma evolution and cancer evolution, confirming that both colorectal adenomas and CRC were of monoclonal origin (47). Davel analyzed scRNA-seq data of 2824 cells from CRC cancer tissues, dividing different cells of tumor tissues into five clusters according to specific genes, further analyzing the cluster data, gene ontology terms, KEGG pathways and trajectory maps.The study found that cluster 1 was characterized by a unique set of genes, such as *IGLC7, IGLC2, IGLC3*, cluster 2 has unique *HLA-DRA, IGHM, IGHG2* other genes, and the remaining three clusters also defined themselves by unique genes. A high degree of specificity between the different clusters was found (48). These results showed that SCS was a powerful tool for studying tumor cell heterogeneity. Owing to the unique high resolution of SCS, scTRIO-SEQ (a type of single-cell triple sequencing) can simultaneously assess somatic copy number changes, DNA methylation, and transcriptomic information and facilitate single-cell heterogeneity research. The high-throughput and high-resolution characteristics of scRNA-seq are also beneficial for the detection of tumor samples (49). Some studies have shown that mutations in *ATM* and *GNAS*, as well as deletions in the tumor suppressor gene *PTEN*, likely led to tumorigenesis because these genes were potential cancer driver genes. Besides, it had been suggested that mutations in *TP53*, *ERBB2*, and *APC* may play an important role in tumorigenesis and may serve as drug targets (50). Markers for two different subtypes of cancer-associated fibroblasts (CAF) were identified using SCS studies. CAF-B cells expressed markers of myofibroblasts such as *TAGLN, ACTA2*, and *PDGFA*, and CAF-A cells expressed *DCN, MMP2*, and *COL1A2*. Only the CAF-A cells expressed the FAP (fibroblast activa tion protein α). Thus, this indicated that the heterogeneity of CAF may constitute a potential barrier to FAP-directed therapy (51).

**Table 7:** SCS in colorectal cancer Heterogeneity.

Sample	Single cell isolation	Molecular level	Amplification	Achievement	Reference
Fresh tumor and adjacent normal tissues from a patient	manual-controlled pipetting system	DNA	Multiple displacement amplification	Activation of SLC12A5 becomed a potential oncogenic driver event in colon cancer by promoting cell proliferation and inhibiting. apoptosis.	(46)
20 normal single cells, 25 polyp single cells, 20 adenomatous polyps single cells, and 50 cancer single cells .	micromanipulation system	DNA	Agilent SureSelect Platform	New mutations were found in *OR1B1* (GPCR signaling pathway) in adenoma evolution, and *LAMA1* (PI3K-Akt signaling pathway) and *ADCY3* (FGFR signaling pathway) in CRC evolution.	(47)
Cancer tissue obtained 2824 cells from CRC patient with stage III C	10x genomics	RNA	10x genomics	High degree of specificity existed for the genes clustered by five cells in the same tumor tissue.	(48)
1,900 single cells from 12 CRC patients (stage III or stage IV)	/	DNA	scTrio-seq (single-cell triple omics sequencing) technique	The feasibility of reconstructing genetic lineages with single-cell multigroup sequencing, tracking its epigenome and transcriptome dynamics was demonstrated.	(49)
9 tumor regions and 88 single cells from two rectal cancer patients	FACS	DNA	multiplexed single-cell MALBAC	Dominant subclones adapted to the surrounding microenvironment played a dominant role in a certain region of a given tumor, and their dominance changed dynamically, such as *TP53*, *ERBB2*, and *APC.*	(50)
590 cells from 11 primary CRC tumors and matched normal samples	10x genomics	RNA	10x genomics	The pathway alteration and diversity of CAFs in CRC and the treatment barriers caused by CAF heterogeneity.	(51)

### SCS Enables the Discovery of the Features of Tumor Microenvironment in CRC

SCS facilitates the discovery of cellular features in the CRC microenvironment (**Table 8**). SCS data revealed that the proportion of somatic copy number alteration (SCNA) in cancer tissues was much higher than that in adjacent normal tissues (11.1% v.s.10.6%), and five genes (*BGN*, *RCN3*, *TAGLN*, *MYL9*, and *TPM2*) were identified as fibroblast-specific biomarkers of poor CRC prognosis. Thus CRC successfully confirmed the extensive genomic alteration in cells in CRC tumor microenvironment (52). Studies of the CRC tumor microenvironment using SCS revealed cancer type-specific T cell subsets and developmental patterns, as well as detailed molecular characterization of tumor immune-related T cell clusters. The cellular and molecular mechanisms underlying the tumor immune microenvironment composition, heterogeneity, and formation were revealed (53). Comprehensive analysis of the non-epithelial scRNA-seq data derived from precancerous lesions and CRC revealed that the proportion of CD8^+^ T cells, natural killer cells, and γδT cells (labeled cytotoxic cells) was significantly increased in serrated polyps compared to that in adenomas (54). Another study examined TAMs in CRC and found that Bcl9 deficiency caused macrophage polarization inhibition from M0 to M2 and altered the CRC tumor microenvironment to further interfere with the inflammation of M0 and M1, the cell type balance and transcription differences in TAMs regulated by BCL9-driven Wnt signaling affected immune surveillance and inflammation in cancer (55). Together, with the support of new technologies, SCS has greatly promoted a thorough understanding of the tumor microenvironment.

**Table 8:** SCS in microenvironment of colorectal cancer.

Sample	Single cell isolation	Molecular level	Amplification	Achievement	Reference
Peripheral blood from 8,982 immune cells of 12 patients with CRC	FACS	DNA	Multiplexed single-cell MALBAC	*BGN, RCN3, TAGLN, MYL9*, and *TPM2* were identified as fibroblast-specific biomarkers with a poor prognosis in colorectal cancer.	(52)
T cells from a total of 12 CRC patients in 4 MSI and 8 MSS patients	/	RNA	SMART-seq2	The CRC-specific T cell subpopulation, included Th17 (CD4_C08-IL23R), follicular T helper cells (CD4_C06-CXCR5), follicular T regulatory cells (CD4_C11-IL10), CD8_C05-CD6, and CD8_C06-CD160. The latter two clusters highly expressed CD69 and ITGAE.	(53)
Pre-cancers and CRCs, serrated polyps (SERs) consisting of hyperplastic polyps (HPs)	TruXTRAC FFPE microTUBE DNA Kit-Column Purifification kit	RNA	ScRNA-seq	The proportion of CD8+ T cells, natural killer cells, and γδT cells (labeled cytotoxic cells) were significantly increased in serrated polyps compared to that in adenomas.	(54)
12 tumor samples from mice model	Magnetic-activated cell sorting (MACS)	RNA	10x genomics	The macrophages interacted with T cells through the CCL3-CCR5, CAF1R-CSF1 and ICAM1-ITGAL to change the T-cell functions in hsBCL9_CT_-24 treated group. Depletion of *Bcl9* maked the CSF1R-CSF1 and CCL4-CCR5 was significantly regulated.	(55)

### SCS Facilitates the Diagnosis and Treatment of CRC

Many researchers have used SCS to make great achievements in the diagnosis and treatment of CRC (**Table 9**). Some studies demonstrated that primary tumor cells evolved for a long time and acquired many mutations such as in *KRAS*, *NRAS*, *APC*, and *TP53*, which spread to distant sites and organs by using high-throughput single-cell DNA sequencing to study the advanced transmission of model metastatic CRC. This transmission model could be extended to many human cancers with important clinical significance (56). Lei et al. conducted scRNA-seq analysis on immune and stromal populations from CRC patients and identified specific macrophage and conventional dendritic cell subsets as key mediators of cellular cross-talk in the tumor microenvironment. Besides, they determined that anti-CSF1R treatment preferentially depleted macrophages with inflammatory features, and CD40 agonist antibody treatment preferentially activated the conventional dendritic cell population (57).

**Table 9:** SCS in diagnosis and treatment of colorectal cancer.

Sample	Single cell isolation	Molecular level	Amplification	Achievement	Reference
Frozen primary colon cancer and matched metastatic CRC in liver tissues	FACS	DNA	High-throughput single-cell DNA sequencing method	APC was the first blow to trigger colon cancer before the *KRAS* and *TP53* mutations. Late transmission indicated that the primary and metastatic tumors had most of the clinically relevant gene mutations( amplification of oncogenes including *CDX2, CDK8, JAK3* and *ZNF217* ).	(56)
Cells were extracted from tumors, adjacent normal tissues, and blood from 18 initially treated CRC patients	FACS	RNA	SMART-seq2	A method to dissect specifically the effects of tumor-associated immune populations was demonstrated.	(57)

## Summary and Prospects

The incidence and mortality rate of malignant tumors in China are the highest among the world, and the overall situation of GI cancer prevention and treatment are very grim. SCS has become an important technique for studying GI cancer. Currently, with its development and integration with other technologies, further improvements and advances in SCS technologies will improve its applicability in clinical settings. However, the technique has some limitations. For example, biological noise will lead to the change of single cell sequencing data and affect the results of data (36). Another limitation is RNA leakage. It may occur during reverse transcription and then may introduce substantial bias (58). SCS also has some shortcomings. Single-cell sequencing is very sensitive to samples and therefore not suitable for analysis of preserved or poorly processed clinical samples.So it is difficult to translate the results from sequencing studies into the clinic (59). The high of SCS cost limits the ability to analyze a large number of tumors, and often only a few to dozens of samples are analyzed per study.

With the development of single-cell multiplexed technologies and the miniaturization and automation of SCS instruments, these limitations and shortcomings may be solved gradually and SCS will have more vast applications in GI cancer research. What’s more, with continuous innovation and optimization of methods, the SCS technology will continue to promote the development of biomedicine and the accurate treatment of GI cancer and may likely aid in achieving high-quality long-term survival for patients with GI cancer.

## Author Contributions

YJ and KL participated in the design, conception. ZX, JL, PH, YZ and JY wrote the review. ZX, JL, YJ and KL revised the review. All authors contributed to the article and approved the submitted version.

## Funding

This work was supported by the National Natural Science Foundations of China (No. 81872335), National Natural Science Youth Foundation (No. 81902486), Technology Innovation Leading Talents (No. 224200510015), The Central Plains Science and Technology Innovation Leading Talents, the Science and Technology Project of Henan Province (No. 212102310187). The National Undergraduate Innovation Experiment Program of Zhengzhou university. This work is supported by The Pathophysiology Department, School of Basic Medical Sciences, College of Medicine, Zhengzhou University.

## Conflict of Interest

The authors declare that the research was conducted in the absence of any commercial or financial relationships that could be construed as a potential conflict of interest.

## Publisher’s Note

All claims expressed in this article are solely those of the authors and do not necessarily represent those of their affiliated organizations, or those of the publisher, the editors and the reviewers. Any product that may be evaluated in this article, or claim that may be made by its manufacturer, is not guaranteed or endorsed by the publisher.

## Abbreviations

CAF, cancer-associated fibroblasts; CNVs, copy number variations; CRC, colorectal cancer; CTC, circulating tumor cells; EC, esophagealcancer; EAC, esophageal adenocarcinoma; FIR, fractionated irradiation; GAC, gastric adenocarcinoma; GC, gastric cancer ; GI, Gastrointestinal; HDGC, hereditary diffuse gastric cancer; IL-17, interleukin-17; LCTCsmulti, large multiploid CTCs; paclitaxel-R, paclitaxel-resistant KYSE-30 cells; PC, pancreatic cancer; PD-1, Programmed cell death protein 1; PC, peritoneal carcinomatosis; scRNA-seq, single-cell RNA sequencing; SCS, single-cell sequencing; SCTCstri, small CTCs with trisomy 8; SPEM, Spasmolytic polypeptide-expressing metaplasia; TAMS, tumor-associated macrophages; Tregs, regulatory cells; WES, whole-exome sequencing.
